# Microglial over-pruning of synapses during development in autism-associated SCN2A-deficient mice and human cerebral organoids

**DOI:** 10.21203/rs.3.rs-3270664/v1

**Published:** 2023-09-28

**Authors:** Yang Yang, Jiaxiang Wu, Jingliang Zhang, Xiaoling Chen, Zhefu Que, Kyle Wettschurack, Brody Deming, Maria acosta, Ningren Cui, Muriel Eaton, Yuanrui Zhao, Manasi Halurkar, Mandal Purba, Ian Chen, Tiange Xiao, Matthew Suzuki, Chongli Yuan, Ranjie Xu, Wendy Koss, Dongshu Du, Fuxue Chen, Long-Jun Wu, Mayo Clinic

**Affiliations:** Purdue University College of Pharmacy & Purdue Institute for Integrative Neuroscience (PIIN); Purdue University College of Pharmacy & Purdue Institute for Integrative Neuroscience (PIIN); Purdue University College of Pharmacy & Purdue Institute for Integrative Neuroscience (PIIN); Purdue University College of Pharmacy & Purdue Institute for Integrative Neuroscience (PIIN); Purdue University College of Pharmacy & Purdue Institute for Integrative Neuroscience (PIIN); Purdue University College of Pharmacy & Purdue Institute for Integrative Neuroscience (PIIN); Purdue University College of Pharmacy & Purdue Institute for Integrative Neuroscience (PIIN); Purdue University College of Pharmacy & Purdue Institute for Integrative Neuroscience (PIIN); Purdue University College of Pharmacy & Purdue Institute for Integrative Neuroscience (PIIN); Purdue University College of Pharmacy & Purdue Institute for Integrative Neuroscience (PIIN); Purdue University College of Pharmacy & Purdue Institute for Integrative Neuroscience (PIIN); Purdue University College of Pharmacy & Purdue Institute for Integrative Neuroscience (PIIN); Purdue University College of Pharmacy & Purdue Institute for Integrative Neuroscience (PIIN); Purdue University College of Pharmacy & Purdue Institute for Integrative Neuroscience (PIIN)

**Keywords:** SCN2A, Microglia, Synaptic pruning, Organoids, Development

## Abstract

Autism spectrum disorder (ASD) is a major neurodevelopmental disorder affecting 1 in 36 children in the United States. While neurons have been the focus to understand ASD, an altered neuro-immune response in the brain may be closely associated with ASD, and a neuro-immune interaction could play a role in the disease progression. As the resident immune cells of the brain, microglia regulate brain development and homeostasis via core functions including phagocytosis of synapses. While ASD has been traditionally considered a polygenic disorder, recent large-scale human genetic studies have identified *SCN2A* deficiency as a leading monogenic cause of ASD and intellectual disability. We generated a *Scn2a*-deficient mouse model, which displays major behavioral and neuronal phenotypes. However, the role of microglia in this disease model is unknown. Here, we reported that *Scn2a*-deficient mice have impaired learning and memory, accompanied by reduced synaptic transmission and lower spine density in neurons of the hippocampus. Microglia in *Scn2a*-deficient mice are partially activated, exerting excessive phagocytic pruning of post-synapses related to the complement C3 cascades during selective developmental stages. The ablation of microglia using PLX3397 partially restores synaptic transmission and spine density. To extend our findings from rodents to human cells, we established a microglial-incorporated human cerebral organoid model carrying an *SCN2A* protein-truncating mutation identified in children with ASD. We found that human microglia display increased elimination of post-synapse in cerebral organoids carrying the SCN2A mutation. Our study establishes a key role of microglia in multi-species autism-associated models of *SCN2A* deficiency from mouse to human cells.

## INTRODUCTION

Autism spectrum disorder (ASD) is a major neurodevelopmental disorder that currently affects 1 in 36 children in the United States [1]. Children with ASD display repetitive behaviors and social deficits, often accompanied by severe comorbidities including intellectual disability with impaired learning and memory [2]. While ASD has been traditionally considered a polygenic disease [3], large-scale human genetic studies have identified a growing number of monogenic causes of ASD [4]. In particular, recent whole genome sequencing studies in humans have demonstrated that loss-of-function and protein-truncating mutations in *SCN2A* (collectively referred to as *SCN2A* deficiency) as a leading monogenic cause of ASD [4–6].

*SCN2A* encodes the voltage-gated sodium channel Nav1.2, a major sodium channel expressed in the central nervous system (CNS) mediating the action potential initiation, propagation, and backpropagation [6–8]. Nav1.2 is widely expressed in the brain including principal excitatory neurons of the cortex, striatum, and hippocampus, but has limited expression in non-neuronal cells [9–11]. Because of the leading role of Nav1.2 deficiency in autism, we recently generated a *Scn2a*-deficient mouse model [12]. These mice display major behavioral and neuronal phenotypes [12, 13], suggesting the utility of this mouse model to study disease pathogenesis.

While neurons have been the focus to understand ASD, an altered immune response in the brain has been suggested to be closely associated with ASD, and a neuro-immune interaction could play a role in the disease progression [14, 15]. In the brain, microglia are the resident immune cells. Microglia regulate brain development and homeostasis via core functions including phagocytosis of synapses, a key structure mediating neuronal communications [16–19]. As an example, microglia contribute to neuronal plasticity during development by engulfing immature spines, leading to the maturation of neurons and their synapses [20, 21]. Moreover, it is also reported that microglia are involved in several mouse models of neurodevelopmental disorders including Fragile X syndrome (Fmr1-KO mice) [22] and Mecp2-null mice of Rett syndrome [23]. As Fmr1 and MECP2 are transcriptional/translational factors important for both neuron and non-neuronal cells [24, 25], it is thus not known how microglia respond in ASD-related disease models in which neurons are the primary cell types affected by the disease-causing genetic mutations.

*Scn2a* is not only a top ASD gene, but is also largely a neuron-specific gene with minimal expression in non-neuronal cells like microglia [26]. Taking advantage of this, here we study multi-species disease models related to *Scn2a* deficiency and investigate how microglia respond in a system where disease-causing genes predominantly affect neurons. We found that *Scn2a*-deficient mice have impaired learning and memory, accompanied by reduced excitatory postsynaptic current (EPSC) and lower synapse density in the neurons of the hippocampus. Utilizing *in vitro* primary culture, we demonstrate that the reduction of synapse density is a microglia-dependent process. Immunolabeling studies further reveal that microglia in *Scn2a*-deficient mice are partially activated with excessive phagocytic pruning of post-synapses related to the complement C3 cascades. The ablation of microglia using PLX3397 during the development reverses EPSC and spine density toward the WT level. To extend our findings to human cells, we established a human induced pluripotent stem cells (hiPSCs) derived cortical organoid model carrying an *SCN2A* protein-truncating mutation C959X identified in children with autism. We found that human microglia exert increased elimination of post-synapse in cortical organoids carrying the C959X genetic mutation, consistent with our finding in the *Scn2a*-deficient mouse model. Together, our study establishes a key role of microglia in multi-species autism-associated models from mouse to human cells.

## RESULTS

### Scn2a- deficient mice display impaired learning and memory.

**Figure 1:**
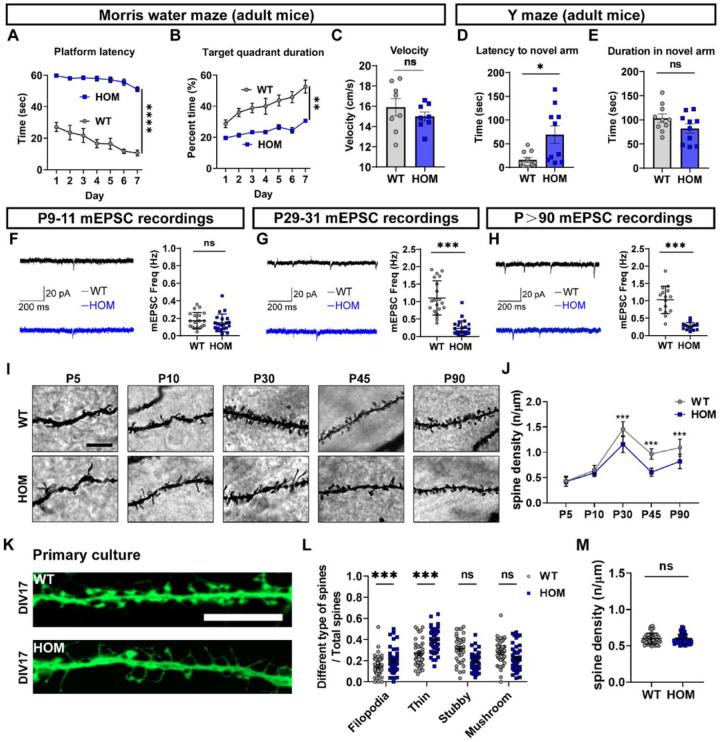
Adult *Scn2a*-deficient (HOM) mice exhibit impaired learning and memory as well as impaired synaptic functions and structures. (**A-C**) Morris water maze: (**A**) Platform latency (time to find the hidden platform) plotted against training day. (**B**) Target quadrant duration (time in the platform quadrant) plotted against training day. Repeated-measures two-way ANOVA. (**C**) Swimming velocity of each group. Two-way ANOVA, n=8 mice for each genotype. (**D-E**) Y maze: (**D**) Latency to novel arm (time to enter the novel arm) of each group. (**E**) Duration in the novel arm (time in the novel arm) of each group. Two-way ANOVA, n=10 mice for each genotype. (**F-H**) Representative images of mEPSCs recorded and quantitation of mEPSCs frequency in hippocampal CA1 pyramidal neurons during the development of P9–11 (**F**), P29–31 (**G**), and P>90 (**H**). Scale bars, 20 pA (vertical) and 200 ms (horizontal). Unpaired *student’s t-test*, P9–11: WT, n=20 cells/4 mice; HOM, n=20 cells/4 mice. P29–31: WT, n=20 cells/4 mice; HOM, n=20 cells/4 mice. P>90: WT, n=12 cells/3 mice; HOM, n=12 cells/3 mice. (**I**) Representative images of Golgi-stained apical dendrites of hippocampal CA1 pyramidal neurons at P5, P10, P30, P45, and P90. Scale bar: 10 μm. (**J**) Quantitation of the spine density of apical dendrites of hippocampal CA1 neurons. Unpaired *student’s t-test*, P5: WT, n=40 cells/5 mice; HOM, n=40 cells/5 mice. P10: WT, n=40 cells/5 mice; HOM, n=40 cells/5 mice. P30: WT, n=40 cells/5 mice; HOM, n=40 cells/5 mice. P45: WT, n=43 cells/4 mice; HOM, n=38 cells/4 mice. P90: WT, n=48 cells/6 mice; HOM, n=48 cells/6 mice. (**K**) Representative images of dendrites from the AAV-CaMKII virus transfected hippocampal primary cultured neurons from WT or HOM mice cultured for 17 days. (**L**) Quantitation of different types of spines. Unpaired *student’s t-test*, WT, n=42 cells from 6 mice, HOM, n=44 cells from 6 mice. (**M**) Quantitation of spine density of hippocampal primary cultured neurons. Unpaired *student’s t-test*, WT, n=42 cells from 6 mice; HOM, n=44 cells from 6 mice. Results are presented as mean ± standard error of the mean (SEM). *p*<0.05 (*), *p*<0.01 (**),*p*<0.001 (***), and *p*<0.0001 (****), ns means not significant.

Profound autism is often accompanied by intellectual disability manifested as impaired cognitive learning and memory [2, 27]. To understand the impact of *Scn2a* deficiency on the learning and memory of mice, the Morris water maze was used to test long-term learning and memory. In the Morris water maze, mice were trained to escape water by remembering the location of a hidden platform. Over the seven-day testing period, WT mice found the platform with an increased performance each day and spent more time in the target quadrant area of the platform (Fig. 1A, B). Meanwhile, *Scn2a*-deficient mice took a significantly longer time to find the platform (often timing out) with an only slight improvement in each subsequent day (Fig. 1A, B). The swimming velocity, however, was similar between WT and *Scn2a* deficient mice, suggesting no significant impairments in swimming ability (Fig. 1C). The Morris water maze results thus reveal a significant impairment of long-term learning and memory in the *Scn2a*-deficient mice.

Additionally, we further assess the shorter-term spatial working memory of *Scn2a*-deficient mice with the Y maze [28]. This test consists of two phases. For the learning phase, mice are trained with one of the arms closed off. Then four hours later, the memory phase of the test was performed with all arms open. It is expected that WT mice should readily explore the previously closed-off “novel” arm. Indeed, we found that WT mice quickly enter the novel arm, while *Scn2a*-deficient mice have a significant delay to enter the novel arm (Fig. 1D), even though the duration in the novel arm was not notably altered (Fig. 1E). Together, our data indicate that *Scn2a-deficient* mice have disrupted learning and memory.

### The synaptic function and structure are impaired in Scn2a-deficient mice.

Impaired learning and memory are often associated with altered synaptic transmission in the hippocampus [29, 30]. To investigate whether Nav1.2 deficiency affects synaptic transmission in principal pyramidal neurons of the hippocampal CA1 region, we recorded miniature excitatory postsynaptic currents (mEPSCs) at several time points during the development (Fig. 1F–H). We found that the frequency of mEPSCs was significantly decreased in *Scn2a*-deficient mice compared to WT mice at the P29–31 and adulthood (P > 90) periods (Fig. 1G, H), but not at the P9–11 developmental period (Fig. 1F). These results suggest an impairment in the synaptic transmission in Nav1.2 deficiency mice starts only in the middle of the developmental stage.

Functional synaptic transmission needs proper pairs of pre- and post-synapses. Thus, it is important to determine whether the number of co-localized pre- and post-synapses markers are altered in *Scn2a*-deficient mice. Since EPSC represents excitatory synaptic transmission, we investigated the excitatory synapses first. To this end, we performed immunohistochemistry (IHC) to detect colocalized VGLUT1 (presynaptic marker) and PSD95 (post-synaptic marker) puncta, as well as Synapsin1/2 (pre-synaptic marker) and Homer1 (post-synaptic marker) puncta (Supplementary Fig. 1A-J). Indeed, we found that the number of co-localized pre- and post-synaptic markers for excitatory synapses in the hippocampus was significantly reduced in P30 and P > 90 with no detectable changes at P5 and P10 (Supplementary Fig. 1A-E, F-J). These results obtained via IHC are consistent with our electrophysiological findings. Additionally, we checked the co-localization of inhibitory synapses with VGAT (pre-synaptic marker) and Gephyrin (post-synaptic marker). Interestingly, we found that inhibitory synapses of hippocampal neurons in *Scn2a*-deficient mice only reduce at P > 90 but display no detectable change at other time points (Supplementary Fig. 1K-O).

Functional alteration in synaptic transmission and reduced pre- and post-synapse colocalization are likely to be accompanied by the change in spine density. To test this, we performed Golgi staining at multiple time points to study the synaptic structure. Consistent with our EPSC and pre- and post-synapse colocalization results, we found that the spine density of hippocampal CA1 decreased significantly from P30 to P > 90 in *Scn2a*-deficient mice, but not at P5 and P10 (Fig. 1I, J). Moreover, we performed spine type analysis and found that hippocampal neurons from *Scn2a*-deficient mice have more immature spines with increased filopodia and thin spines in both apical and basal spines during many of the development stages (P5, P10, and P30) *in vivo* (Supplementary Fig. 2A-F). Notably, we also found that the mature spines (e.g., mushroom spines) decreased during most of the developmental stages we examined. Our data thus suggest that while neuronal dendritic spines in hippocampal CA1 of *Scn2a*-deficient mice are less mature throughout development starting from P5, the spine density only starts to decrease at around P30 and continues to be low into adulthood.

Spine density in neurons could be controlled by neuron intrinsic machinery but may also be dynamically regulated by other cell types including microglia *in vivo* [19]. To dissect these possibilities, we established primary neuron culture from *Scn2a*-deficient mice and WT littermates to study the spine density and spine maturity of hippocampal primary neurons without microglia (Fig. 1K–M). We found that the proportion of immature spines (e.g., filopodia and thin spines) of neurons from *Scn2a*-deficient mice increased substantially *in vitro* (Fig. 1L), similar to what we found *in vivo*. Interestingly, we did not find a significant difference in the spine density of hippocampal primary neurons between the WT group and the *Scn2a*-deficient group *in vitro* (Fig. 1M), which is different from our *in vivo* results. Together, these results are likely to suggest that the Nav1.2 deficiency *per se* mainly affects the maturity of the dendritic spine but not spine density in hippocampal pyramidal neurons. Another cell type in the brain (but not in the *in vitro* culture) thus could be essential to modulate the spine density in neurons of *Scn2a*-deficient mice *in vivo*.

### The hippocampal microglia have altered morphology and display an increased volume of lysosome in adult Scn2a-deficient mice.

**Figure 2:**
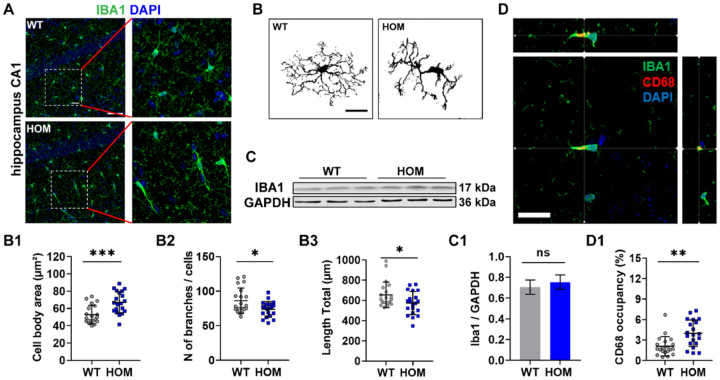
Hippocampal microglia in *Scn2a*-deficient (HOM) mice exhibit altered morphology and increased volume of the lysosome (CD68 marker). (**A**) Representative images of Iba1 labeled hippocampal microglia from WT or HOM mice. Scale bar: 20 μm. Left two panels scale bar: 40 μm, right two panels scale bar: 20 μm. (**B**) Representative microglial morphology of each group. (**C**) Representative Western blot of Iba1 and GAPDH from each group. (**D**) Representative orthogonal image of hippocampal microglia from adult *Scn2a*-deficient mice. Each cross in the three graphs represents the CD68 marker labeled microglial lysosome from three angles. Scale bar: 20 μm. (**B1**) Quantitation of microglial cell body area from each group. Unpaired *student’s t-test*, WT, n=20 cells/5 mice; HOM, n=20 cells/5 mice. (**B2**) Quantitation of the number of microglial branches from each group. Unpaired *student’s t-test*, WT, n=20 cells/5 mice; HOM, n=20 cells/5 mice. (**B3**) Quantitation of the total length of the microglial process from each group. Unpaired student’s t-test, WT, n=20 cells/5 mice; HOM, n=20 cells/5 mice. (**C1**) Quantitation of Iba1 expression compared to GAPDH between samples from WT or HOM mice. Unpaired *student’s t-test*, WT, n=3 mice; HOM, n=3 mice. (**D1**) Quantitation of CD68 positive occupancy (%) from each group. Unpaired *student’s t-test*, WT, n=20 cells/5 mice; HOM, n=20 cells/5 mice. Results are presented as mean ± standard error of the mean (SEM). *p*<0.05 (*), *p*<0.01 (**), and *p*<0.001 (***), ns means not significant.

Microglia, as the resident immune cells and the primary phagocytes in the brain, play a key role in regulating neuronal plasticity and participate in the pathogenesis of neuronal diseases by excessively engulfing synapses (pre-synapses or post-synapses) [31–33]. Our finding of no notable difference in spine density between the WT and the *Scn2a*-deficient neurons *in vitro* and reduced spine density *in vivo* prompted us to hypothesize that microglia may be responsible for the reduced spine density in *Scn2a*-deficient mice. We thus further studied the morphology and gene expression of microglia *in vivo* in our disease model (Fig. 2A–C). We found that the microglial morphology was changed, with increased cell body area (Fig. 2B1), decreased number of branches (Fig. 2B2), and reduced total length of the process (Fig. 2B3) in hippocampal CA1 of adult (P>90) *Scn2a*-deficient mice compared to adult WT mice. It seems that this change in microglia morphology is not limited to the hippocampal region as we found similar changes in microglial morphology in the medial prefrontal cortex in our *Scn2a*-deficient mouse model (Supplementary Fig. 3A-D). Microglial morphological changes are often accompanied by microglial activation, neuroinflammation, or proliferation [34]. Interestingly, the Western blot result showed that there was no significant difference in Iba1 protein expression between WT and *Scn2a*-deficient groups (Fig. 2C, C1), suggesting minimal changes in microglia proliferation. However, the volume of microglial lysosome marker (CD68) increased significantly in the Scn2a-deficient group compared to the WT group (Fig. 2D, D1). The elevated CD68 positive volume data indicates that microglia are likely to be partially activated, exerting increased phagocytosis. Taken together, our morphological analysis and immunolabeling results indicate that microglia are partially activated in *Scn2a*-deficient mice.

### Excessive phagocytic pruning of synapses by microglia occurs during development and adulthood in Scn2a-deficient mice.

**Figure 3:**
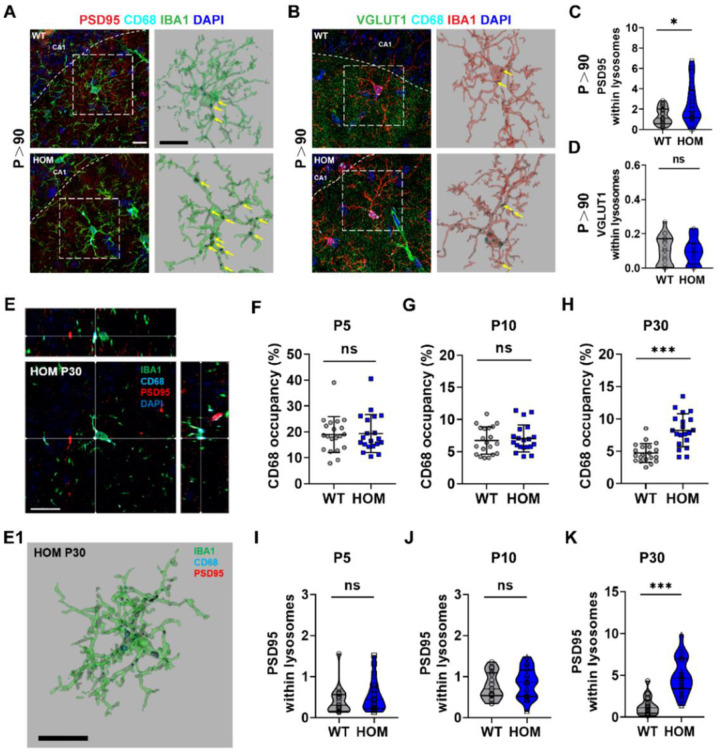
Excessive phagocytic pruning of microglia occurs during development and continues into adulthood in the hippocampus of *Scn2a*-deficient (HOM) mice. (**A-B**) Representative images of post-synapse engulfment (**A**) and pre-synapse engulfment (**B**) by microglia in the hippocampus from each group. Scale bar: 10 μm. (**C, D**) Quantitation of post-synapse engulfment (**C**) and pre-synapse engulfment (**D**) by hippocampal microglia from each group. Unpaired *student’s t-test*, WT, n=20 cells/5 mice; HOM, n=20 cells/5 mice. (**E**) Representative orthogonal image of hippocampal microglia from P30 *Scn2a*-deficient mice. Each intersection point in the three graphs represents the PSD95 post-synaptic marker engulfed within microglial lysosome marker CD68 from three angles. Scale bar: 20 μm. (**F-H**) Quantitation of CD68 positive occupancy (%) from each group at P5 (**F**), P10 (**G**), and P30 (**H**). Unpaired *student’s t-test*, P5: WT, n=20 cells/5 mice; HOM, n=20 cells/5 mice. P10: WT, n=20 cells/5 mice; HOM, n=20 cells/5 mice. P30: WT, n=20 cells/5 mice; HOM, n=20 cells/5 mice. (**E1**) Representative image of hippocampal microglia from P30 *Scn2a*-deficient mice using Imaris for 3D reconstruction. Scale bar: 20 μm. (**I-K**) Quantitation of post-synapse engulfment by hippocampal microglia from each group at P5 (**I**), P10 (**J**), and P30 (**K**). Unpaired *student’s t-test*, P5: WT, n=20 cells/5 mice; HOM, n=20 cells/5 mice. P10: WT, n=20 cells/5 mice; HOM, n=20 cells/5 mice. P30: WT, n=20 cells/5 mice; HOM, n=20 cells/5 mice. All results were presented as mean ± SEM. *p*<0.05 (*), *p*<0.001 (***), ns means not significant.

Since we identified partial microglia activation in adult *Scn2a*-deficient mice, and phagocytosis of spines is a primary function of activated microglia [35], we further investigate whether the activation of microglia translates into enhanced synaptic phagocytosis in the *Scn2a*-deficient mice. Moreover, if they do, which kind of synapses (pre- vs post-synapses) do the microglia prefer to engulf? To this end, we assessed microglial engulfment of pre-synapses (VGLUT1) and post-synapses (PSD95) in hippocampal CA1 of adult mice. We performed IHC for VGLUT1 or PSD95 tri-stained with IBA1 (microglia staining) and CD68 (lysosome staining) (Fig. 3A, B). We quantitated the amount of VGLUT1 or PSD95 within IBA1^+^/CD68^+^ microglial lysosomes. We found that microglial engulfment of PSD95 post-synapses was significantly elevated in adult *Scn2a*-deficient mice, but display no detectable changes in engulfment of VGLUT1 pre-synapses (Fig. 3C, D). These data show that the phagocytosis of microglia in the hippocampus has been elevated and has a selective over-engulfment of post-synapses to regulate the neuronal plasticity in adult *Scn2a*-deficient mice.

Since we identified a post-synapse specific enhancement of engulfment by microglia in adulthood, we seek to understand when this engulfment starts during the development. To this end, we performed IHC for PSD95 tri-stained with IBA1 and CD68 and examined at three time points (P5, P10, and P30). We choose these time points because, at P5, microglia have not yet entered the hippocampus; At P10, microglia start to enter the hippocampus; and at P30, the number of microglia reaches the peak of the whole life [36]. Using the orthogonal view (Fig. 3E) and the reconstruction images (Fig. 3E1), we confirm that the PSD95^+^ areas were indeed in the volume of IBA1^+^/CD68^+^ labeling from three angles of view at P30 (Fig. 3E). Moreover, we found that the occupancy of CD68^+^ inside IBA1^+^ was significantly increased only at P30 in *Scn2a*-deficient mice, but not at P5 and P10, compared to the WT mice (Fig. 3F–H).

Similarly, we quantitated the amount of PSD95 within IBA1^+^/CD68^+^ microglial lysosomes and found that microglial engulfment of PSD95 synapses was only elevated at P30 in *Scn2a*-deficient mice, but not P5 and P10 (Fig. 3I–K). Representative images of these analyses were shown in (Supplementary Fig. 4A-C). Together, our results indicate that excessive post-synaptic pruning occurs from P30 when the microglia have peaked at the hippocampus and continues during the development into adulthood (P > 90), consistent with our EPSC, pre- and post-synapse co-localization, and spine density results.

### Complement component C3 is involved in the microglial phagocytosis of synapses in Scn2a-deficient mice.

**Figure 4:**
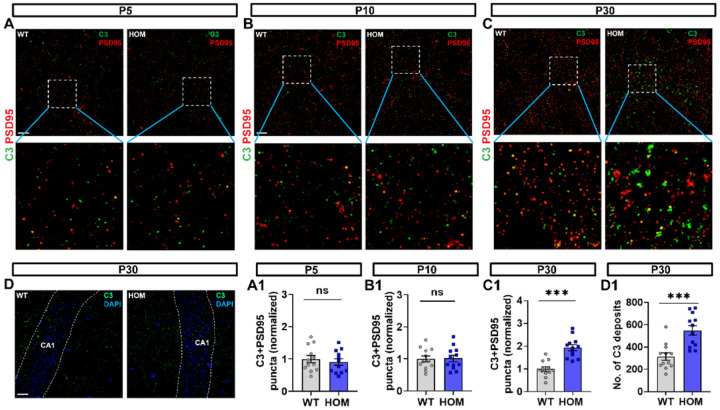
Complement components C3 localized to post-synapses (PSD95) in hippocampal CA1 of *Scn2a*-deficient (HOM) mice during development. (**A**) Representative images of hippocampal CA1 immunolabeled with post-synaptic PSD95 (red) and complement component C3 (green) at P5 from each genotype. Scale bar: 10 μm. (**A1**) Quantitation of colocalized puncta of C3 with PSD95 at P5. Unpaired student’s t-test, n=12/3 mice for both genotypes. (**B**) Representative images of hippocampal CA1 immunolabeled with post-synaptic PSD95 (red) and complement component C3 (green) at P10 from each genotype. Scale bar: 10 μm. (**B1**) Quantitation of colocalized puncta of C3 with PSD95 at P10. Unpaired *student’s t-test*, n=12/3 mice for both genotypes. (**C**) Representative images of hippocampal CA1 immunolabeled with post-synaptic PSD95 (red) and complement component C3 (green) at P30 from each genotype. Scale bar: 10 μm. (**C1**) Quantitation of colocalized puncta of C3 with PSD95 at P30. Unpaired *student’s t-test*, n=12/3 mice for both genotypes. (**D**) Representative images of hippocampal CA1 immunolabeling of C3 deposits from each genotype. Scale bar: 20 μm. (**D1**) Quantitation of the number of C3 deposits. Unpaired *student’s t-test*, n=12/3 mice for both genotypes. Results were presented as mean ± SEM. *p*<0.05 (*), *p*<0.01 (**), and *p*<0.001 (***), ns means not significant.

The classical complement cascade-dependent phagocytic signaling pathway is a molecular mechanism used by microglia to prune synapses during development [37, 38]. Therefore, we hypothesize that during the development of *Scn2a*-deficient mice, the classical complement cascades pathway could be overactivated and leading to the phagocytosis of a large number of immature dendritic spines. To investigate whether the C3 and C1q target the post-synapses, we performed IHC for colocalized PSD95 and C3 or C1q puncta (Fig. 4A–C, Supplementary Fig. 5A-C). We found that the puncta levels of both PSD95^+^/C3^+^ and PSD95^+^/C1q^+^ in *Scn2a*-deficient mice were significantly increased at P30, but not at P5 or P10 compared to WT mice (Fig. 4A1, B1, C1, Supplementary Fig. 5A1, B1, C1).

Increased co-localization of C3/C1q with PSD95 may result from switched locations of C3/C1q or overly increased C3/C1q deposits. To test this, we performed IHC for C3 and C1q deposits at P30 in hippocampal CA1 from WT and *Scn2a*-deficient mice (Fig. 4D, Supplementary Fig. 5D). We found that both C3 and C1q deposits were elevated in *Scn2a*-deficient mice compared to WT mice (Fig. 4D1, Supplementary Fig. 5D1). These data show that it is likely that during early development, the increased number of immature dendritic spines leads to an overactivation of the complement pathway, producing extra C3 and C1q deposits to promote microglia to engulf excessive immature dendritic spines.

### Synaptic transmission and spine density can be partially restored in hippocampal CA1 of Scn2a-deficient mice with microglia ablation.

**Figure 5:**
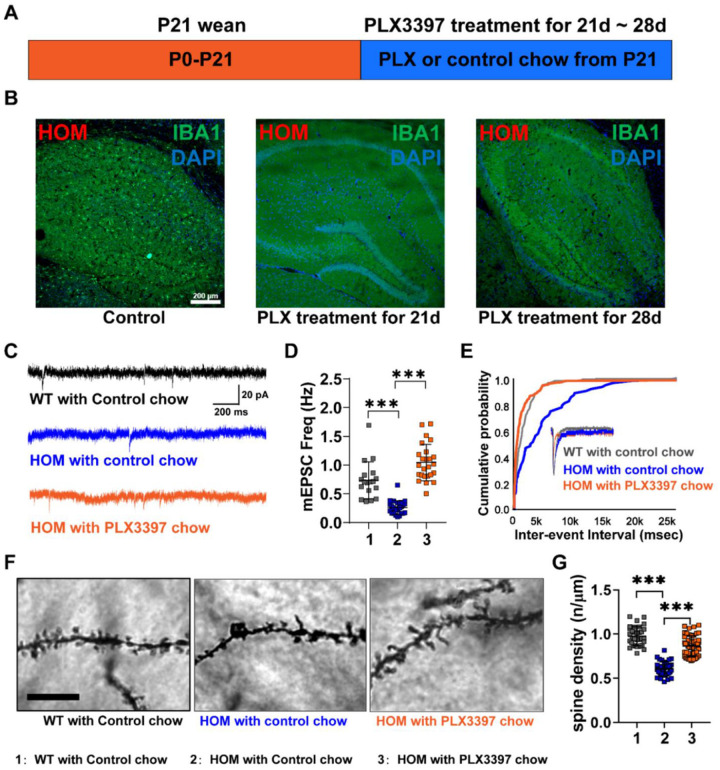
Microglia depletion during development restores neural transmission and spine density of hippocampal pyramidal neurons of *Scn2a*-deficient (HOM) mice. (**A**) Experimental timeline for microglial depletion in mice during the development. (**B**) Representative images of Iba1 staining in the hippocampus of control P45 HOM mice (left panel), PLX3397 treatment for 21 days (middle panel), and PLX3397 treatment for 28 days (right panel). Scale bar: 200 μm. (**C**) Representative mEPSCs recordings of hippocampal CA1 pyramidal neurons from each group. Scale bars, 20 pA (vertical) and 200 ms (horizontal). (**D**) Quantitation of mEPSCs frequency from each group. *One-way ANOVA*, WT with control chow: n=18 cells/3 mice; HOM with control chow: n=25 cells/5 mice; HOM with PLX3397 chow: n=25 cells/5 mice. (**E**) Associated cumulative probability of each group. (**F**) Representative images of Golgi-stained apical dendrites of hippocampal CA1 pyramidal neurons from each group. Scale bar: 10 μm. (**G**) Quantitation of the spine density of apical dendrites of hippocampal CA1 neurons from each group. *One-way ANOVA*, WT with control chow: n=33 cells/4 mice; HOM with control chow: n=38 cells/4 mice; HOM with PLX3397 chow: n=60 cells/5 mice. Results were presented as mean ± SEM. *p*<0.001 (***).

To further investigate a causal role of microglia in synapse functions, we next studied whether microglia ablation from early life would restore altered synaptic functions. Microglia depletion was initiated using the CSF1R inhibitor PLX3397 formulated in the chow for consecutive 3–4 weeks starting from P21 (Fig. 5A). Efficient ablation of microglia from the hippocampus was achieved within three weeks (Fig. 5B). Whole-cell patch-clamp recordings were then performed with hippocampal CA1 pyramidal neurons of *Scn2a*-deficient mice with control chow or PLX3397 chow (Fig. 5C). We found that the frequency of mEPSCs was increased in neurons from *Scn2a*-deficient mice treated with PLX3397, accompanied by a large shift of its cumulative curve, compared to *Scn2a*-deficient mice treated with control chow (Fig. 5D, E). Notably, the frequency of mEPSCs in *Scn2a*-deficient mice with PLX3397 chow was increased to almost the same level as that in WT mice with control chow (Fig. 5D, E). As a positive control, we found that WT mice with PLX3397 chow had a higher frequency of mEPSCs than WT mice with control chow, which is consistent with published studies [39] (Supplementary Fig. 6A-C). Together, our data indicate that microglia depletion could partially restore the altered synaptic transmission in *Scn2a*-deficient mice.

As microglia participate in the synaptic remodeling and plasticity by altering spine density [38, 40, 41], we next examined the spine density in control and PLX3397-treated *Scn2a*-deficient mice. We found that the spine density was also partially restored in *Scn2a*-deficient mice with PLX3397 chow compared to the *Scn2a*-deficient mice with control chow (Fig. 5F, G), showing a significant increase towards the WT value. These results further confirm that microglia play a key role in regulating synaptic functions, and microglia depletion could partially restore synaptic transmission and spine density in *Scn2a*-deficient mice.

### Human microglia exert enhanced elimination of post-synapses in human cortical organoids carrying autism-associated SCN2A-C959X mutation.

**Figure 6:**
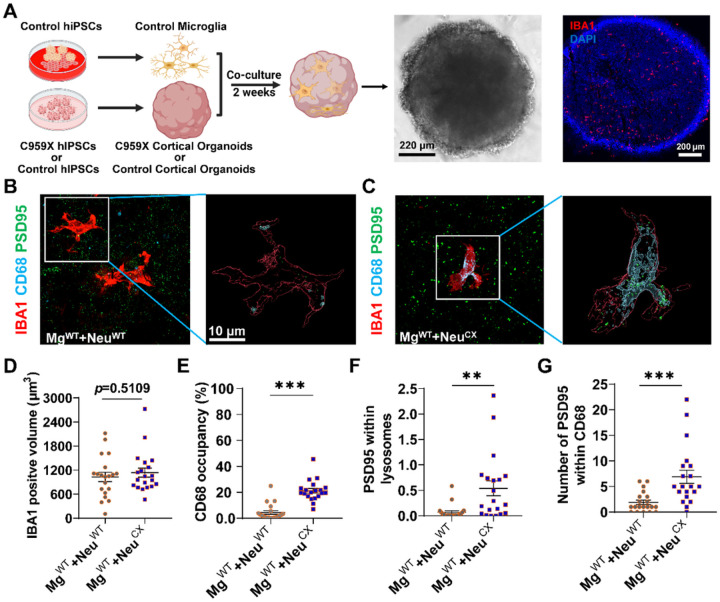
Increased post-synapse elimination by hiPSC-derived microglia in cortical organoid carrying autism-associated *SCN2A*-C959X mutation. (**A**) Schematic representation of developing microglia-containing cortical organoids and representative brightfield image and Iba1 staining image. Brightfield image: scale bars, 220 μm, Iba1 staining image: scale bars, 200 μm. (**B-C**) Representative triple-staining images (Iba1, PSD95, and CD68) and Imaris reconstructed images from control microglia co-cultured with the control cortical organoids (**B**) or C959X cortical organoids (**C**). Scale bar, 10 μm. (**D**) Quantitation of Iba1 labeled microglial volume from each group. Unpaired t-test, Mg^WT^+Neu^WT^, n=20 cells/5 organoids, Mg^WT^+Neu^CX^, n=20 cells/5 organoids. (**E**) Quantitation of CD68 positive occupancy (%) from each group. Unpaired *student’s t-test*, Mg^WT^+Neu^WT^, n=20 cells/5 organoids; Mg^WT^+Neu^CX^, n=20 cells/5 organoids. (**F**) Quantitation of post-synapse engulfment by control microglia from each group. Unpaired *student’s t-test*, Mg^WT^+Neu^WT^, n=20 cells/5 organoids; Mg^WT^+Neu^CX^, n=20 cells/5 organoids. (**G**) The number of PSD95 puncta inside CD68 labeled microglial lysosome from each group. Unpaired student’s t-test, Mg^WT^+Neu^WT^, n=20 cells/5 organoids; Mg^WT^+Neu^CX^, n=20 cells/5 organoids. Results are presented as mean ± standard error of the mean (SEM). *p*<0.05 (*), *p*<0.01 (**), and *p*=0.5109 means not significant.

Autism-associated *SCN2A* loss-of-function and protein-truncating mutations result in *Scn2a* deficiency, which could partially be modeled with the *Scn2a*-deficient mouse model. To extend our findings from rodent models to human cells, we established a human-induced pluripotent stem cells (hiPSCs) model carrying a heterozygous *SCN2A* protein-truncating/stop-codon C959X mutation. Heterozygous C959X mutation was identified in patients with autism, and the C959X mutation in *SCN2A* leads to a non-functional Nav1.2 channel (*SCN2A* deficiency) [42]. To study the C959X mutation, we engineered it into the reference iPSCs [43, 44] accordingly to our published protocols [45, 46]. These engineered human iPSCs then were differentiated into cerebral organoids that are expected to partially recapitulate the complexity of the human brain. Using this advanced human cell-based model, we aim to study how microglia regulates synapses in human neurons with Nav1.2 deficiency. Since it is known that Nav1.2 is minimally expressed in the microglia [26], we deliberately only generated human microglia from the reference (control/WT) iPSCs to simplify the research design. Once generated, control hiPSCs-derived microglia would then be added to the cortical organoids (C959X or control cortical organoids) for two weeks (Fig. 6A). Excitingly, we found that the human microglia could grow uniformly, incorporate, and distribute evenly within C959X organoids as well as control cortical organoids (Fig. 6A). We then performed IHC for PSD95 tri-stained with IBA1 and CD68 to understand whether the human microglia may respond to C959X organoids or control organoids differently (Fig. 6B, C). We reconstructed the 3D images of both types of organoids for quantification. While we did not notice a notable change in the size of microglia (IBA1 positive volume) (Fig. 6D), interestingly we found that the CD68 occupancy (within IBA1 + area) was significantly increased in human microglia co-cultured with C959X organoids compared to these co-cultured with control organoids (Fig. 6E). Moreover, we revealed that both volumes of PSD95^+^ positive signals within CD68^+^ (Fig. 6F) and the number of PSD95^+^ puncta within CD68^+^ (Fig. 6G) were significantly elevated in human microglia co-cultured with C959X organoids, consistent with what we found in the *Scn2a*-deficient mice. Together, our results from the human cells-based model further support that microglia have enhanced synaptic phagocytosis in neurons with *SCN2A* deficiency, extending our findings from rodent models to human cells.

## DISCUSSION

In this present study, we found that autism-associated *Scn2a*-deficient mice showed impaired learning and memory accompanied by decreased dendritic spine density and miniature excitatory postsynaptic currents (mEPSCs). We further revealed that microglia are partially activated to regulate neuronal synaptic plasticity by engulfing immature post-synapses during development, which continues into adulthood. Using PLX3397 to ablate microglia, we show that dendritic spine density and the frequency of mEPSCs were partially restored towards the WT level. To translate our findings obtained in rodent models to human cells, we generated a three-dimensional (3D) microglia-containing cerebral organoid model by utilizing human iPSCs carrying the *SCN2A*-C959X mutation identified in children with autism. With this advanced human cell-based organoids model, we revealed that excessive synaptic pruning of microglia also occurs when co-cultured with neurons carrying autism-associated *SCN2A*-C959X mutation. Taken together, we provide multi-species evidence using rodent models and human cells to suggest that the synaptic pruning functions of microglia are crucial to regulate synaptic transmission and modulate synapse density in autism-associated disease models of *SCN2A* deficiency.

ASD is traditionally viewed as a polygenic disease, heavily influenced by gene-environment interaction [47, 48]. However, recent large-scale whole genome/exome sequencing studies have identified a growing portion of profound autism with a monogenic cause [4]. Among them, *SCN2A* has been revealed to be a top gene on the list with an “infinite” odds ratio, suggesting a 100% penetration [2]. As *SCN2A* is predominantly expressed in the neurons with minimal expression in the non-neuronal cells [26], the prior studies of *SCN2A*-related autism have been largely focused on the neurons [9, 49–52]. Here in this current study, we show for the first time, that even if *SCN2A* has minimal expression in non-neuronal cells, microglia can respond to the diseased neurons with *SCN2A* deficiency and modulate spines and synaptic transmission *in vivo* in mice and *in vitro* in human cerebral organoids. Our study thus suggests that the expression of disease-associated genes in microglia is not a prerequisite for the involvement of microglia in disease pathogenesis. Rather, dysfunctional neurons *per se* could trigger the responses of microglia, much like microglia respond to external factors like traumatic brain injuries [53]. While this finding is not extremely surprising, prior studies with genetic models of autism mainly involve the study of genes that are expressed in both neurons and microglia [18, 22, 23, 54, 55], preventing such a conclusion from being clearly drawn.

Recently human studies have suggested the involvement of the immune system in psychiatric disorders like ASD [56, 57]. Ongoing immune dysregulation is noted in the ASD populations [15], and it is further found that microglia are activated in young adults with ASD [14]. As a resident immune cell in the brain, microglia can exert multiple functions including phagocytosis of synapses, damaged neurons, or pathogens. They could also release cytokines to mediate local inflammation response, as well as maintain the microenvironmental homeostasis of the brain. Among the many core functions of microglia, our current study is mainly focused on the microglia phagocytosis of synapses, which is prompted by our finding of impaired spine density and synaptic transmission. It is possible that microglia may display altered cytokines release and promote inflammation [34, 58, 59]. For example, it is found in the maternal immune activation (MIA) mouse model related to autism, microglia were activated and secreted cytokines and prostaglandins to recruit astrocytes, exacerbating the inflammatory response [60, 61]. We did not pursue this direction as our initial study of cytokines-related protein expression did not reveal notable differences in our *Scn2a*-deficient mice (data not shown). However, it is worth pointing out that our focus is on the hippocampal and cortical brain regions. We currently cannot exclude whether other types of immune activation may be triggered by microglia in other brain regions of our *Scn2a*-deficient mouse models.

It is interesting to note that in this study, we found that microglia selectively over-prune the post-synapses, evident by enhanced colocalization of post-synaptic markers like PSD95 with microglia lysosome marker CD68. Microglia can regulate dendritic spine density by either engulfing presynaptic or postsynaptic elements [62–70]. In particular, it was found that microglia mainly prune postsynaptic markers in a mouse model of translation initiation factor (eIF4E) overexpression, resulting in autism-like behaviors [67]. This study is consistent with our findings. However, another study reveals that microglia selectively prune pre-synaptic markers in a mouse model of demyelinating disease models related to multiple sclerosis (MS) [62]. It is currently unknown why microglia mainly prune post-synapses in our autism-associated *Scn2a*-deficient mice and human cell models. The complement cascade (including C1q and C3), among other signaling pathways, has been identified as key mediators in phagocytosis and pruning of synapses by microglia during development [35, 38, 71–74]. Because we found an increased number of C1q and C3 targeting the post-synapses, we suspect that the selective over-pruning of post-synapses in *Scn2a*-deficient disease models by microglia could be directed by the positions of C1q and C3 molecules. It is suggested that C3 is mainly released by astrocytes [75] while C1q can be secreted by activated microglia [76, 77]. We also found an increase in the population of astrocytes (data not shown). Since activated astrocytes can produce an increased number of C3, it is possible that the overproduction of C3 together with C1q released by microglia labels the immature post-synapses, mediating microglial engulfment. Thus, the involvement of astrocytes and potential microglia–astrocyte interaction could be a possible underlying mechanism. Further studies are needed to elucidate the detailed molecular pathway underlying microglia phagocytosis and microglia-astrocyte interaction.

The human iPSCs-based model with human genetic background has now emerged as an advanced system to study human diseases [78]. Since microglia are originating from the yolk sac [79], the strategy to generate microglia is distinct from that to generate neurons [80, 81]. A co-culture model thus is needed to study how microglia interact with neurons. Indeed, studies have demonstrated the feasibility of generating a microglia-containing neuronal model to provide a more physiologically relevant *in vitro* model [82, 83]. For example, it has been shown that microglia are able to carry out normal phagocytosis functions inside midbrain organoids to increase neuron maturation and functionality [82]. It was also shown that the microglia cultured *in vitro* could respond to physical disruptions of neuronal cells by displaying ameboid morphologies after a transient integration into the brain organoids [80]. Additionally, the microglia-organoid model was used to study neurodegenerative disorders [81, 83, 84]. This current study, to our knowledge, is the first one to utilize the microglia-cortical organoids model to study disease-causing mutation related to ASD. Using this three-dimensional microglia-containing cortical organoid model, we demonstrated that the microglia can be successfully integrated into the neuronal organoids and display ramified morphology, suggesting that the microglia in the organoid have preserved a relatively homeostatic microglia state. Importantly, we found that the hiPSC-derived microglia integrated into the neuronal organoid retained a functional synaptic pruning ability. In human cortical organoids carrying *SCN2A*-C959X mutation, we found that microglia display an increased PSD95 engulfment and lysosomal upregulation, largely recapitulating our findings in the mouse models. It is also worth noting that in the prior studies, myeloid progenitors or primitive macrophage progenitors were incorporated into premature organoids to promote the co-development of microglia and neurons [85, 86]. However, the long-term culture of organoids may develop necrotic inner layers due to hypoxia [87], which may cause unexpected microglial reactions. To focus on the microglial pruning functions in our disease model, we cultured microglia separately from the organoids, and then incorporate the microglia with organoids at a later stage. This approach opens the opportunity to understand the transient microglial reaction to diseased neurons. This advanced human microglia-organoids co-culture model could also be used as a promising tool to model brain development and other neurological or psychiatric disorders.

In summary, here in this paper, we reported a comprehensive study to understand the role of microglia in disease models of *SCN2A* deficiency related to ASD. We revealed that microglia play a major role in the disease pathogenesis of autism-associated *Scn2a*-deficient mouse models via excessive phagocytosis of post-synapses, even though *Scn2a* is minimally expressed in microglia. The ability of microglia to prune excessive synapses of diseased neurons seems to be evolutionarily conserved, observed in both rodent and human cell models of *SCN2A* deficiency. Our study underscores the importance to have a holistic view to study neurodevelopmental/psychiatric disorders like ASD, highlighting the need to take neuron-immune interactions into consideration.

## MATERIALS AND METHODS

### Animals.

C57BL/6N-*Scn2a*^*1tm1aNarl*^/Narl (referred to as *Scn2a*^*WT/gt*^) mice were generated from the National Laboratory Animal Center Rodent Model Resource Center based on a modified gene-trap design [88, 89]. This mouse model’s generation and primary characterization are available in our recent articles [12, 52]. All mice were maintained at 22°C on a 12-h/12-h light/dark cycle with ad libitum access to water and food. Heterozygous (HET, *Scn2a*^*WT/gt*^) mice were used as breeding pairs to obtain homozygous (HOM, *Scn2a*^*gt/gt*^) mice and WT littermates for study. Adult mice (85–95 days old) and developing mice (1–50 days old) of both sexes were used. All animal work was approved by the Institutional Animal Care and Use Committee (IACUC).

### Behavior tests.

The Y-maze is a two-phase spatial reference/working memory task [28] that uses a plastic apparatus with three arms of 5 cm wide × 35 cm long with 20 cm tall transparent walls placed 120° apart (Maze Engineers). Black shapes (dots, stripes, and squares) were positioned at the end of each arm to serve as cues. The training phase consists of blocking a random arm and placing the mouse at the end of an unblocked arm, facing the cue. After 5 min of exploration, each mouse was placed back in its home cage. Four hours later, the retention phase was conducted, during which the mouse was placed in the same spot, with all the arms open, and recorded for 5 min. The latency to the novel arm, number of novel arm entries, duration in the novel arm, and alternations were recorded using EthoVision XT. The Y-maze was cleaned with 70% isopropanol/water solution before and after each animal and wiped dry to reduce social cues.

The Morris water maze measures learning and memory through training to escape water by swimming to a hidden platform using visual cues. The circular pool was 130 cm in diameter and 81 cm tall (Maze Engineers, Boston, MA, USA) and filled with 21 ± 1°C water mixed with washable white paint to make it opaque. Water was fresh every day. The platform was submerged 1–1.5 cm below the water’s surface Pretraining was performed the day before the first day of testing to reduce the effects of stress on task performance. During pretraining, mice were placed in the tank at different points along the wall with the platform in the middle. If they did not find the platform after 60 seconds, they were guided to it and sat there for 15 seconds before being dried off with a microfiber cloth. Days 1–7 of testing consisted of four trials per day (30 minutes in between trials) with the platform in the middle of the northwestern quadrant (“target quadrant”), where it remained for the duration of the experiment. The mice were dropped in a different direction for each trial (south, west, north, or east). Similar to the pretraining, if they did not find the platform after 60 seconds, they were guided to it and sat there for 15 seconds before being dried off with a microfiber cloth. Latency to the platform, duration in the target quadrant, and velocity were recorded using EthoVision XT (Noldus, Leesburg, VA, USA).

### Electrophysiology.

Mice at different time points (P9–11, P29–31, and P90) were used for electrophysiological recording. As described in our previous article [52], Ketamine/xylazine (100/10 mg/kg, i.p., 0.1 mL per 10 g of body weight.) was used to anesthetize mice deeply and the mice were transcardially perfused. We quickly decapitated the mice’s heads and placed the brain into an ice-cold slicing solution when the organs and limbs of the mice turned white. Acute coronal slices containing hippocampus (300 mm in thickness) were cut with a vibratome (Leica VT1200S, Germany). Before use to patch, the slices were incubated in the same solution for 10 min at 33°C, then transferred to normal artificial cerebrospinal fluid (aCSF) at 33°C for 10–20 min and at room temperature for at least 30 min. Slices were visualized under IR-DIC (infrared-differential interference contrast) using a BX-51WI microscope (Olympus) with an IR-2000 camera (Dage-MTI). All somatic whole-cell patch-clamp recordings were performed from identified hippocampal CA1 pyramidal neurons. The mEPSC of different groups by whole-cell voltage-clamp recordings in the presence of TTX (sodium channel blocker, 0.5 μM), bicuculline (GABA_A_ receptor antagonist, 10 μM), and CGP55845 (selective GABA_B_ receptor antagonist, 2 μM) to block inhibitory synaptic transmission.

### Western blot.

The protein cleavage extraction solution was made using RIPA lysis extraction buffer (Thermo Fisher Scientific, 89901) and protease phosphatase inhibitors (Thermo Fisher Scientific, A32953). The hippocampus tissues were mixed with the protein cleavage extraction solution and homogenized. Nanodrop (Thermo Scientific) was used to measure the protein concentration of each group. Before running the gel, the proteins were boiled at 95°C for 5 min to denature. Proteins were separated by 8% SDS-PAGE and transferred onto PVDF membranes (0.45 μm; Millipore, Billerica, USA). Membranes were placed in 5% nonfat milk in Tris-buffered saline and Tween 20 (TBST) for 30 min at room temperature to block nonspecific binding sites before incubating overnight at 4°C with primary antibody solution. On the second day, TBST was used to wash the membranes for 8 mins/3 times, followed by incubation with corresponding IRDye 680RD secondary antibodies in TBST for 2h at room temperature. After following 8 mins/3 times wash with TBST, the immunoreactive bands were scanned and captured by the Odyssey CLx Imaging System (LI-COR Biosciences) and quantitatively analyzed by densitometry with Image Studio Lite 5.2 (LI-COR Biosciences).

### Golgi Stain.

FD Rapid GolgiStain^™^ Kit (PK401; FD Neurotechnologies, Inc.) was used for Golgi Stain. Briefly, mice were administered an overdose of anesthesia and decapitated to dissect brains into ice-cold PBS solution. Whole brains were sequentially immersed in impregnation solution (mixed with Solutions A and B for 24 hours in advance) after removing the impurities and blood and stored at room temperature for at least 2 weeks. The samples were transferred into Solution C for over 72 hours in the dark, then frozen with dry ice as fast as possible. Cryostat was performed to cut into sections of 100 μm thickness serially. The slices were stained using silver nitrate solution (Solutions D and E) for 10 min, then wash with double distilled water 2 times for 4 min for each rinse. The brain slices were dehydrated in 50% 75% and 95% ethanol solutions for 4 min sequentially, then dehydrated in 100% ethanol solutions 4 times for 4 min for each rinse. Xylene was used to clear the brain slices 3 times for 4 min each rinse. Spine density and morphology were analyzed using Neurolucida software (Neurolucida^®^ 360 - MBF Bioscience).

### Immunofluorescence.

The mice were anesthetized with ketamine/xylazine perfused through the ascending aorta with 20 ml of heparinized saline followed by 20 ml of freshly prepared 4% paraformaldehyde in 0.01 M phosphate-buffered saline (PBS, pH 7.4). Brain tissues were dissected and post-fixed in 4% PFA overnight at 4°C, then placed in 20% sucrose at 4°C until they sunk to the bottom. Afterward, brain tissues were placed in 30% sucrose at 4°C until they sunk to the bottom. Optimal cutting temperature compound (OCT compound, Fisher Scientific, USA) was used to embed tissue samples before frozen sectioning on the cryostat, and Frozen 40-μm coronal sections were cut. Slices were placed into PBS and stored at 4°C for future research. The free-floating stain was performed to increase the staining efficiency. The sections were placed in PBST solution (0.5% TritonX-100 in PBS) to permeabilize for 30 min, then incubated with blocking buffer (0.5%TritonX-100and5%normal goat serum in PBS) for one hour at room temperature. Primary antibodies were the following: anti-Iba1 (cat# ab178846, Abcam), human anti-Iba1 (cat#234308, Synaptic Systems), anti-CD68 (cat# MCA1957, Bio-Rad), human anti-CD68 (cat# MA5–13324, Invitrogen), anti-PSD95 (cat# 124011, Synaptic Systems), human anti-PSD95 (cat# 36233S, Cellsignal), anti-Homer1 (cat# 160003, Synaptic Systems), anti-Synapsin1/2 (cat# 106004, Synaptic Systems), anti-vGlut1 (cat# 135304, SySy), anti-C1q (cat# ab182451, Abcam), anti-C3 (cat# ab11862, Abcam). The primary antibodies were mixed with blocking buffer and incubated the sections overnight at 4°C. The following day, sections were rinsed with 0.01 M PBS and incubated for 2 h with secondary antibodies at room temperature. Finally, coverslips were applied after the sections were rinsed. Confocal images were obtained using Z-stack laser scan confocal fluorescence microscope (LSM900; Carl Zeiss, Jena, Germany). Imaris Cell Imaging Software was performed to analyze the 3D images. The volume of the reconstructed surfaces for IBA1 and CD68 were recorded, and the percentage of CD68 occupancy within microglia was calculated using the following formula: volume of CD68/volume of IBA1 + cell. To quantitate the PSD95^+^ or VGLUT1^+^ within CD68^+^, we used the following formula: PSD95^+^ volume or PSD95^+^ volume/CD68^+^ volume 100.

### Mouse neuronal primary culture.

The primary neuronal culture was performed to detect the spine of cultured hippocampal neurons *in vitro*. Briefly, P0 pups were anesthetized by placing them on ice for 5 minutes. Once cryo-anesthetized, the pups were killed by cervical dislocation and decapitated in ice-cold Hank’s balanced salt solution (HBSS) in a dissection hood. Meninges were cleared and the brain tissues were dissected rapidly with fine scissors and tweezers. Then the hippocampus was isolated under the dissection microscope. The hippocampus tissues were transferred into the cell culture hood when all samples were collected. 0.25% Trypsin-EDTA and DNase1 were used to digest the tissues at 37°C for 15 min. After that, we used the 1-ml serological pipette to dissociate the tissues and washed the cell suspension 3 times with DMEM. Pre-warmed plating medium (Neurobasal A supplemented with B-27, GlutaMax, and penicillin-streptomycin) was added and passed through a 40-μm cell strainer. Cells were grown in the plating medium and maintained at 37°C in 5% CO2. For the immunostaining study, Cytarabine was added into the medium (2.5 μg/mL, half-change) to kill the microglia cells. At 7–9 primary culture days, the AAV-CaMKII virus was added to viral transfection for 10 days.

### Microglia ablation.

To deplete microglia, the mice were treated with chow containing colony-stimulating factor 1 receptor (CSF1) inhibitor PLX3397 (600 mg/kg, Chemgood company) and control chow, starting from postnatal 21 for 4 weeks. The special chow was made by Research Diets Inc. [90]. Immunostaining was performed to assess the Efficacy of microglial ablation at selected time points.

### Human cortical organoid generation and culture.

CRISPR/Cas9 edited human induced pluripotent stem cells (hiPSCs) expressing WT (Control) and C959X *SCN2A* mutation were used to create cortical organoids using the published protocol developed[91]. The hiPSCs colonies were maintained daily in StemFlex Medium (Gibco, Cat#. A334940) and aggregated at a cell density of 100cells/μL in Essential 8 medium (Gibco, Cat#. A1517001) to form spheroidal embryoid bodies (EBs). Round bottom Ultra-low-attachment plates (Corning Costar, Cat#. CLS7007) and centrifugation at 100g for 3 minutes aided the formation of EBs overnight. The EBs were maintained in Essential 6 media (Gibco, Cat#. A1516401) for the first six days, supplemented with activin/nodal/TGF-β and BMP pathway inhibitors Dorsomorphin (DM) and SB-4321542 [92] and 1.25 μM XAV-939 for neuronal induction via DUAL-SMAD method. EBs were harvested and transferred to flat-bottomed 6 well-formatted suspension culture plates (Corning, Cat# 347) and maintained in Neurobasal-A media with Glutamax (Gibco, Cat# 35050061) and Penstrep (10000U/mL) prophylactic (Gibco, Cat# 15140163), B27 minus A supplement (Gibco, Cat# 12587010) with 20 ug/mL Human Recombinant FGF2 (Fibroblast growth factor 2) and 20 ug/mL EGF (Epidermal growth factor) with complete medium changes every day for 17 days followed by every two days until day 23. Afterward, the medium supplements were replaced with 20 ng/mL BNDF (Brained Derived Neurotrophic Factor), 20 ng/mL NT3 (Neurotrophin-3), 50 μM cAMP (Cyclic adenosine monophosphate), 10 μM DHA (cis-4,7,10,13,16,19-Docosahexaenoic acid) and AA (L-ascorbic acid (AA) 2-phosphate trisodium salt) and changed every two days to achieve organoid differentiation until day 46. After that, the organoids will be maintained in Neurobasal–A basal medium, B27 + supplement with no growth factors until day 150, with media changes every 4–5 days.

### Human microglia differentiation from iPSCs.

The differentiation of microglia largely follows previously published protocol [81]. Briefly, iPSCs were differentiated into hematopoietic progenitor cells following the instructions from a commercially available kit (STEMdiff Hematopoetic kit) for 12 days. Then the progenitors were passaged into the microglia differentiation medium, with DMEM/F12, 2× insulin-transferrin-selenite, 2× B27, 0.5× N2, 1× non-essential amino acids, 400 μM monothioglycerol, and 5 μg/mL human insulin. Immediately before use, the microglia differentiation medium were supplemented with 100 ng/mL IL-34, 50 ng/mL TGF-β1, and 25 ng/mL M-CSF for up to 24 days.

### Microglia integration in the mature cortical organoid.

To promote the integration of microglia into the organoids, the organoids (matured for more than 120 days) were individually placed and transferred into the ultra-low attachment 96-well plate. Then, the day 12 microglia were harvested and seeded with the density of 400,000 cells per well on top of the organoids in the fresh culture medium containing half microglia differentiation medium and half mature cortical organoid medium. The spontaneous integration of microglia into organoids was allowed for 7 days, with daily medium change. Then the organoids were transferred back into the ultra-low attachment 6-well plate and continued to grow for another 7 days according to the general cortical organoid maintenance protocol. Then, organoids were fixed with 4% PFA for the following immunostaining.

### Statistical analysis.

Origin Pro, GraphPad Prism 8, Adobe Illustrator CC, Imaris, Neurolucida360, Image J, miniAnalysis, and Clampfit were used for data analysis and curve fitting. Two-tailed Student’s t-test (parametric) or unpaired two-tailed Mann-Whitney U-test (non-parametric) was used for single comparisons between two groups. The other data were analyzed using one-way or two-way ANOVA and then using a post hoc with Bonferroni corrections. The number of experimental samples (n) in each group was indicated in the legend. Results are presented as mean ± standard error of the mean (SEM). Significance was determined when *P* < 0.05 (*), *P* < 0.01 (**), and *P* < 0.001 (***).
